# Longitudinal Associations of Psychiatric Risk Factors with Non-psychiatric Hospitalization in a Large Cohort of People Living with HIV in New York City

**DOI:** 10.1007/s10461-023-04064-6

**Published:** 2023-04-21

**Authors:** Aaron S. Breslow, Melissa Fazzari, Peter J. Franz, David B. Hanna, Uriel R. Felson, Elizabeth Cavic, Marla R. Fisher, Laurie Bauman

**Affiliations:** 1 PRIME Center for Health Equity, Department of Psychiatry and Behavioral Sciences, Albert Einstein College of Medicine, Bronx, NY USA; 2 Psychiatry Research Institute at Montefiore Einstein, Department of Psychiatry and Behavioral Sciences, Albert Einstein College of Medicine, Bronx, NY USA; 3 Einstein-Rockefeller-City University of New York Center for AIDS Research, Bronx, NY USA; 4 Department of Psychiatry, Icahn School of Medicine, Mount Sinai Morningside and Mount Sinai West, New York, NY USA; 5 Departments of Pediatrics and Psychiatry and Behavioral Sciences, Albert Einstein College of Medicine, Bronx, NY USA; 6 PRIME Center for Health Equity, Albert Einstein College of Medicine, 1300 Morris Park Avenue, Van Etten 4A-47, Bronx, NY 10461 USA

**Keywords:** Mental health, Hospitalization, Depression, HIV/AIDS

## Abstract

Hospitalizations among people living with HIV (PLWH) are frequent and costly. This study examined the association between psychiatric, HIV-related, and demographic factors and hospitalization rates among PLWH using data from the Einstein–Rockefeller-City University of New York Center for AIDS Research Clinical Cohort Database. Of the 10,215 PLWH included in the sample, 45% had at least one non-psychiatric hospitalization between 2009 and 2018, with significant risk factors including prior psychiatric outpatient visits, depression, or alcohol-related disorder diagnoses, female sex, older age, CD4 count < 500 cells/uL, and detectable viral load. Additionally, 14% had an HIV-related hospitalization, with significant risk factors including prior psychiatric outpatient visits, alcohol- and substance-related disorder diagnoses, female sex, older age, CD4 count < 500 cells/uL, and detectable viral load. The study emphasizes the need for tailored interventions, including integrated treatment and comprehensive case management, for PLWH with comorbid psychiatric disorders, women, and older adults.

**Supplementary Information:**

The online version contains supplementary material available at 10.1007/s10461-023-04064-6.

## Resumen

Las hospitalizaciones son frecuentes y costosas entre las personas que viven con VIH (PVVIH). Este estudio examinó la asociación entre factores psiquiátricos, relacionados con el VIH y demográficos, y las tasas de hospitalización en 10,215 PVVIH. Entre 2009 y 2018, el 45% de los pacientes tuvieron al menos una hospitalización no psiquiátrica. Los factores de riesgo significativos incluyeron más visitas previas a la consulta psiquiátrica ambulatoria, diagnóstico previo de depresión o trastorno relacionado con el alcohol, sexo femenino, edad avanzada, conteo de células CD4 < 500 células/uL, y carga viral detectable. De las 10,215 PVVIH, el 14% tuvo una hospitalización relacionada con el VIH. Los resultados destacan la necesidad urgente de apoyo dirigido a PVVIH con trastornos psiquiátricos comorbilidades, y para mujeres y adultos mayores que viven con VIH.

Hospitalizations among people living with HIV (PLWH) are frequent, avoidable and costly, yet also present a critical opportunity for medical and behavioral health intervention [1–3]. Annual rates of hospitalizations among PLWHA in the United States range from 27 to 34% depending on geographical location, year, and various social and structural determinants of health [4]. With continued improvements in efficacy, safety profiles, and convenience (i.e., single tablet formulations) of antiretroviral therapy (ART) in the last decade, rates of HIV-related hospitalizations and mortality have significantly decreased [3–5]. In the United States, for example, the number of HIV-related deaths fell by half from 2010 to 2017 [6]. In New York State, HIV-related hospitalizations declined 48% between 2009 (12,444) and 2017 (6492). [7] Yet disparities persist; HIV-related hospitalizations and mortality remain disproportionately high for people of color (particularly Black PLWH) and those with psychiatric comorbidities [3, 4, 8]. This is concerning given the cost and burden of frequent hospitalizations, as well as the toll of frequent, acute crises for PLWH [9, 10]. There may be underutilized intervention opportunities to keep PLWH with psychiatric disorders out of the hospital, or to bolster support during hospitalizations [4, 5].

In this study, we aimed to identify psychiatric risk factors for hospitalization, including number of outpatient psychiatric visits prior to hospitalization, and types of psychiatric diagnoses in a patient’s chart prior to hospitalization. Little is known about psychiatric predictors of hospitalization, particularly for non-psychiatric hospitalization among PLWH. Medical drivers are well-documented [5], with the primary risks for hospitalization in the United States including severe non-AIDS comorbidities, e.g., cardiovascular, renal and liver diseases [3]. Indeed, PLWH often describe a ‘treatment paradox,’ wherein nonadherence to ART leads to illness, yet long-term ART use may exacerbate chronic inflammation and immunological abnormalities [11]. Though lifesaving, long term ART use may also cause lipodystrophy and long-term toxicity, driving need for inpatient care among older PLWH and those with more clinically significant HIV-related indicators of health [10].

There also may be disparities in terms of associations between psychiatric health and hospitalizations, and intersecting demographic factors, among diverse cohorts of PLWH [12]. Psychiatric health impacts HIV-related outcomes in profound ways, and mental health concerns often occur in the context of HIV stigma, minority stressors [13] and structural determinants of health [14, 15]. These forces drive psychiatric risk for adverse HIV-related outcomes among women, older adults, and people of color [17, 18]. Physiologically, both HIV and ART impact (often gendered) inflammatory processes which may cause neuro-psychiatric impairments such as dementia, brain atrophy, and encephalitis [11, 16, 17]. Psychiatric comorbidities often go untreated, or are treated through siloed, versus integrated, care and in emergency or inpatient settings [12, 18]. Although disproportionately high for PLWH, little is known about how psychiatric risk factors may influence need for inpatient care [1, 2].

## The Present Study

To this end, we measured associations of psychiatric, demographic, and HIV-related factors with any non-psychiatric or HIV-related hospitalization. Data are from the Einstein-Rockefeller-City University of New York Center for AIDS Research (ERC-CUNY) Clinical Cohort Database, a clinical database described below. We tested three hypotheses: Hypothesis 1: Patients with more past visits to treat a psychiatric disorder will have a higher hazard for any non-psychiatric hospitalization, and HIV-related hospitalization in particular. Hypothesis 2: Patients with depression, anxiety, drug-related diagnoses and alcohol-related diagnoses will have a higher hazard for hospitalization than those without these diagnoses. Hypothesis 3: Patients with more significant HIV-related factors (i.e., CD4 count < 200 cells/uL, detectable viral load) and certain demographic factors (i.e., older, female, Black or Hispanic) will have a higher hazard of hospitalization.

In doing so, we aimed to identify particular risk factors for hospitalization, in order to prevent inpatient stays earlier in patients’ care continua, and/or make better use of hospitalizations as an opportunity for secondary prevention [2, 3]. Results may hold important implications for services, echoing calls for comprehensive case management for PLWH and comorbid psychiatric diagnoses [19], and for behavioral support for PLWH.

## Methods

### Study Design and Populations

We conducted a retrospective cohort study of all patients with a laboratory-confirmed diagnosis of HIV and > 1 visit at Montefiore Health System (Montefiore), an integrated health care delivery system with 4 hospitals and > 50 ambulatory care sites and one of the oldest wraparound HIV centers in the United States [20]. Montefiore is located primarily in the Bronx, New York: the poorest of the five boroughs in New York City and the poorest of the 62 counties in the state, as well as a major epicenter of the ongoing HIV/AIDS epidemic, with a prevalence of 2.0–2.5% compared with 1.5% in New York City and 0.4% nationally [21]. Montefiore provides HIV services across a network of primary care centers and outpatient substance treatment programs, an infectious disease clinic, a sexual health clinic, and an adolescent program.

Data for this paper come from the ERC-CFAR Clinical Cohort Database, which contains clinical data on patients living with HIV and receiving care within Montefiore [14, 22]. This database is based primarily on electronic health records (EHR) of a racially diverse cohort of PLWH receiving care in the Bronx, New York. It combines clinical data such as outpatient and inpatient visits, laboratory test results, and prescription data with behavioral information from New York State’s AIDS Institute Reporting System (AIRS). For this study, we included data on adult patients with confirmed HIV infection with > 1 visit to a Montefiore site from January 1, 2009 through December 31, 2018 [23]. Patients who were hospitalized at their index visit were excluded from analysis.

### Outcomes of Interest

The outcomes of interest were time to the first one of two types of hospitalization: any non-psychiatric (i.e., medical) hospitalization; and HIV-related hospitalization in particular. Hospitalizations were classified based on diagnosis codes, operationalized as International Classification of Diseases, Ninth Revision, Clinical Modification (ICD-9-CM) codes, through 2015 or International Classification of Diseases, Tenth Revision, Clinical Modification (ICD-10-CM) codes, 2015 and later, assigned at discharge. Non-psychiatric hospitalizations and HIV-related hospitalizations were not mutually exclusive. HIV-related hospitalizations were coded as a particular subset of non-psychiatric hospitalization and analyzed in a separate model. Both outcomes were analyzed as time-to-event outcomes from a patient’s index visit, their first visit in the database (i.e., the first date with any diagnosis recorded), or January 1, 2009 if they received care prior to the study period.

### Primary Exposures of Interest

The primary exposure of interest was psychiatric comorbidity. Specifically, we identified two psychiatric factors as exposures: the number of outpatient psychiatric visits prior to hospitalization; and the type of psychiatric diagnoses in patients’ charts. For the first factor, we counted the number of outpatient psychiatric visits prior to incident hospitalization, to capture potential associations between outpatient psychiatric services utilization and time to incident inpatient visit. Outpatient visits were classified as psychiatric based on the assignment of an ICD-9-CM code (ranging from 290 to 319) or ICD-10-CM code (ranging from F01-F99) for treatment of a mental, behavioral, or neurodevelopmental disorder.

For the second factor, we classified patients as having a particular type of psychiatric diagnosis if they had at least one visit prior to incident hospitalization assigned an ICD-9-CM or ICD-10-CM code indicating treatment of: alcohol related disorder, substance related disorder, anxiety, and/or depression. These were not mutually exclusive, and patients were often classified as having multiple types of psychiatric diagnoses. See Table S1 in supplement for the coding scheme.

### Confounders and Other Variables

We extracted additional patient-level characteristics to account for potential confounding by demographic or HIV-related factors. Demographic factors included patient age (in years), sex assigned at birth (female/male), and race/ethnicity (White non-Hispanic, Black non-Hispanic, Hispanic, and other). HIV-related factors included CD4 T-cell count (< 200, 200–349, 350–499, 500 + cells/uL, or missing at entry), and viral load (undetectable, detectable, or missing at entry). Viral load was classified as undetectable based on the assay used. Most commonly, a threshold of < 40 copies/mL was used in this analysis.

### Statistical Analysis

First, we calculated descriptive statistics (i.e., frequencies, medians, and interquartile ranges) to describe the characteristics of the sample. Second, we examined differences in non-psychiatric and HIV-related hospitalizations by psychiatric, demographic, and HIV-related factors, using a cause-specific calendar-time stratified Cox proportional-hazards regression model with a counting process approach [24]. In doing so, we estimated the associations of psychiatric factors (i.e., number of previous psychiatric visits; type of psychiatric diagnosis), demographic factors [i.e., age, sex assigned at birth (male, female), race and ethnicity], and HIV-related factors (CD4 count, viral load) with the time from index visit to each type of hospitalization. Death was treated as a censoring event.

Each outcome was examined separately. For both outcomes, we examined each variable first as a singular predictor in the model using unadjusted analyses. All models were then adjusted for all other variables, as well as stratified by calendar period (2009–2012, 2013–2015, 2016–2018), to allow for differences in the baseline hazard of hospitalization (e.g., changes in HIV-related care, healthcare policies, and delivery systems that may have occurred within the time frame of interest). Because multiple exposures and confounders were time-varying (i.e., number of previous psychiatric visits, types of psychiatric diagnoses, CD4 count, viral load), we defined each as the most recent value prior to or at the start of the risk interval. This allowed us to update the value of these predictors when new visits occurred, or new laboratory results were available. Hazard ratios, 95% confidence intervals and corresponding p-values for the model coefficient were generated. Trend tests were computed via statistical contrasts using a Cox proportional-hazards regression model, assuming a linear relationship between an ordinal predictor and hospitalization risk. The consistency of the observed effects within each calendar period was examined through the addition of interaction terms in the model and calendar period-specific analyses. Two-sided *p*-values ≤ 0.05 were considered statistically significant. All analyses were completed using SAS version 9.4 (SAS Institute Inc, Cary NC, USA).

### Sensitivity Analysis

Because a large number of patient charts were missing data on CD4 count (*n* = 1091) and viral load (*n* = 1060) within 90 days of their index visit, we imputed missing HIV-related data in two ways [25]. First, we conducted multiple imputation of CD4 count and viral load at patients’ index visit and last observation carried forward (LOCF) if missing CD4 or viral load status at any visit post-entry. For each unique visit date, the closest prior CD4 count and viral load data were identified. If no CD4 count was recorded prior to the index visit, all laboratory values within 90 days of the initial visit date were examined, allowing delays in laboratory measurements and reporting. Second, we conducted complete case (CC) analysis, requiring CD4 count and viral load counts at entry, which removed 1233 patients (12%).

## Results

Between January 1, 2009, and December 31, 2018, among 10,215 eligible patients, 4642 (45%) were hospitalized at least once for a non-psychiatric concern. The median time to incident hospitalization was 30.8 months. Among patients with any non-psychiatric hospitalization, 1,399 (14% of the full sample; and 30% of patients with an incident hospitalization) were hospitalized for an HIV-related concern. The median observation time to first HIV-related hospitalization was 54.5 months.

Table 1 shows demographic and HIV-related characteristics of PLWH. Among the cohort, 58% were male and 42% were female. The mean age was 45 years old. Most patients were racial/ethnic minorities: 43% Black non-Hispanic, 38% Hispanic, 14% other/unknown race, and 5% White non-Hispanic. Among patients with HIV-related laboratory values in their charts within 90 days of their index visit, the largest group (39%) had CD4 count > 500 cells/uL; and most (53%) had a detectable viral load. Of note, 1091 patients were missing documentation of CD4 count within 90 days of their first visit (308/1091 had no CD4 count documented at all) and 1060 were missing documentation of their viral load within those first 90 days (with 294/1060 patients having no viral load documented at all).

**Table 1:** Demographic and HIV-related characteristics of the sample at index visit (*n* = 10,215)

	*n*	%
Demographic factors		
Sex assigned at birth		
Female	4303	42.12
Male	5912	57.88
Race/ethnicity		
White non-Hispanic	485	4.75
Black non-Hispanic	4443	43.49
Hispanic	3907	38.25
Other/Unknown	1380	13.51
Age (in years)—mean (SD) [IQR]	45.4 (12.3) [38–54]	
HIV-related factors		
CD4 T-cell count (cells/uL)		
< 200	1966	21.55
200–349	1810	19.84
350–499	1835	20.11
≥ 500	3513	38.5
Missing CD4 at entry visit^a^	1091	
Viral load undetectable	4317	47.15
Missing viral load at entry visit^a^	1060	

^a^No CD4 or viral loads prior to or within 90 days of initial visit. 308/1091 and 294/1060 patients have no CD4 T-cell counts or viral loads on record at all. The rest have CD4 T-cell count or viral load measurements outside of the 90-day window after the initial appointment.

### Associations with any Non-psychiatric Hospitalization

Table 2 shows the unadjusted and adjusted associations of each factor with any non-psychiatric hospitalization. In unadjusted analyses, patients with either psychiatric factor had an increased hazard for non-psychiatric hospitalization. Specifically, patients were significantly more likely to be hospitalized if they had 1–2 [hazard ratio (HR) 1.16], 3–5 (HR 1.16), 6–10 (HR 1.37), or > 10 (HR 1.46) previous psychiatric visits compared with none (p < 0.0001), and if they had a history of an alcohol-related disorder (HR 1.40, p = 0.006), substance-related disorder (HR 1.35, p < 0.0001), anxiety (HR 1.16, p = 0.01), or depression (HR 1.25, p < 0.0001). In terms of demographic characteristics, patients had significantly higher hazard if they were female (HR 1.25) or older (HR 1.01, both p < 0.0001), and lower hazard if they were other/unknown race (HR 0.74, p = 0.0003) compared with White Non-Hispanic patients. Patients with CD4 count < 500 cells/uL (< 200 cells/uL, HR 3.05; 200–349 cells/uL, HR 1.54; 350–499 cells/uL, HR 1.22, all p < 0.0001) or a detectable viral load (undetectable HR 0.69, p < 0.0001) were also at significantly higher risk, compared to those with CD4 count of 500 or greater or an undetectable viral load.

**Table 2:** Risk factors for any non-psychiatric hospitalization among people living with HIV

Parameter	Unadjusted		Adjusted^a^	
	HR (95% CI)	p value^d^	aHR (95% CI)	p value^d^
Number of outpatient psychiatric visits^b^				
Reference: 0				
1–2	**1.16 (1.08, 1.26)**	0.0001	**1.1 (1.01, 1.19)**	0.04
3–5	**1.16 (1.04, 1.29)**	0.008	**1.11 (0.99, 1.25)**	0.08
6–10	**1.37 (1.21, 1.54)**	< 0.0001	**1.28 (1.12, 1.46)**	0.0004
> 10	**1.46 (1.33, 1.61)**	< 0.0001	**1.39 (1.22, 1.58)**	< 0.0001
			p(trend)^c^ < 0.0001	
Any previous diagnosis^b^ of				
Alcohol-related disorder	**1.40 (1.19, 1.65)**	< 0.0001	**1.21 (1.02, 1.43)**	0.03
Reference: no alcohol-related disorder				
Substance-related disorder	**1.35 (1.24, 1.47)**	< 0.0001	1.05 (0.95, 1.16)	0.30
Reference: no substance-related disorder				
Anxiety disorder	**1.16 (1.07, 1.26)**	0.001	1.00 (0.90, 1.1)	0.96
Reference: no anxiety disorder				
Depression disorder	**1.25 (1.16, 1.34)**	< 0.0001	**1.10 (1.0, 1.19)**	0.05
Reference: no depression disorder				
Assigned female at birth	**1.25 (1.18, 1.33)**	< 0.0001	**1.31 (1.24, 1.39)**	< 0.0001
Reference: male				
Race/ethnicity				
Reference: White Non-Hispanic				
Black non-Hispanic	0.98 (0.85, 1.12)	0.76	0.98 (0.85, 1.13)	0.83
Hispanic	0.94 (0.82, 1.08)	0.36	0.91 (0.79, 1.05)	0.20
Other/Unknown	**0.74 (0.63, 0.87)**	0.0003	**0.78 (0.66, 0.92)**	0.003
Age (in years)	**1.01 (1.01, 1.01)**	< 0.0001	**1.01 (1.01, 1.01)**	< 0.0001
CD4 T-cell count (cells/uL)				
Reference: ≥500				
< 200	**3.05 (2.83, 3.28)**	< 0.0001	**3.01 (2.78, 3.27)**	< 0.0001
200–349	**1.54 (1.42, 1.68)**	< 0.0001	**1.53 (1.4, 1.68)**	< 0.0001
350–499	**1.22 (1.11, 1.33)**	< 0.0001	**1.23 (1.13, 1.34)**	< 0.0001
Viral load undetectable	**0.69 (0.65, 0.73)**	< 0.0001	**0.92 (0.86, 0.98)**	0.011
Reference: detectable				

Bold indicates statistical significance p ≤ 0.05

*HR* hazard ratio, *aHR* adjusted hazard ratio

^a^Model consisting of all Table 2 predictors (number of previous outpatient psychiatric visits, types of previous diagnoses, sex assigned at birth, race/ethnicity, age, CD4 T-cell count, viral load)

^b^Time-varying covariate

^c^Test for linear trend across number of previous psychiatric visits

^d^Corresponding to test of nonzero regression coefficient in the stratified Cox model

In analyses adjusting for all other variables in the model, the hazard of non-psychiatric hospitalization remained significantly higher with any number of previous outpatient psychiatric visits > 0 [1–2 visits, adjusted hazard ratio (aHR) 1.1, 95% CI 1.01–1.19; 3–5 visits, aHR 1.11, 95% CI 0.99–1.25; 6–10 visits, aHR 1.28, 95% CI 1.12–1.46; >10 visits, aHR 1.39, 95% CI 1.22–1.58; p(trend) < 0.0001]. There was no indication that the observed effects due to the number or type of mental health diagnoses changed meaningfully across calendar period. Patients with a history of alcohol-related disorder (aHR 1.21, 95% CI 1.02–1.43, p = 0.03) and/or a depression disorder (aHR 1.10, 95% CI 1.00–1.19, p = 0.05) had higher risk than those without. In terms of demographic characteristics, patients had higher adjusted hazard if they were female compared to male (aHR 1.31, 95% CI 1.24–1.39, p < 0.0001) or older (aHR 1.01 per year, 95% CI 1.01–1.01, p < 0.0001), though lower if their race/ethnicity was Other/Unknown compared to White Non-Hispanic (aHR 0.76, 95% CI 0.66–0.92, p = 0.003). In terms of HIV-related factors, hazard was higher for patients with CD4 counts < 500 cells/uL had higher hazard (e.g., < 200 cells/uL: aHR 3.01, 95% CI 2.78–3.27; 200–349 cells/uL: aHR 1.53, 95% CI 1.40–1.68; 350–499 cells/uL: aHR 1.23, 95% CI 1.13–1.34), as compared to those with CD4 count > 500 cells/uL (all p < 0.0001). Adjusted hazard was significantly higher for those with detectable viral load (undetectable viral load aHR 0.92, CI 0.86–0.98, p = 0.01). Sensitivity analyses did not meaningfully change results. Specifically, after imputing missing HIV-related data using two separate methods, associations of psychiatric, HIV-related, and demographic factors with any non-psychiatric hospitalization remained stable.

### Associations with HIV-Related Hospitalization

Table 3 shows the unadjusted and adjusted associations of each factor with HIV-related hospitalization. Factors significantly associated with HIV-related hospitalization in unadjusted analyses (all p < 0.01) included any number (> 0) of previous psychiatric visits (HRs ranging from 1.71 to 1.88); history of a diagnosis of alcohol-related disorder (HR 2.16, p < 0.0001), substance-related disorder (HR 2.07, p < 0.0001), anxiety disorder (HR 1.21, p = 0.013) or depression disorder (HR 1.20, p = 0.006); CD4 count < 500 cells/uL (< 200 cells/uL, HR 19.18, p < 0.0001; 200–349 cells/uL, HR 4.54, p = 0.0001; 350–499 cells/uL, HR 1.56, p < 0.0001); and detectable viral load (undetectable HR 0.25, p < 0.0001). Age, sex, and race/ethnicity were not significantly associated with this outcome in unadjusted analyses.

**Table 3:** Risk factors for HIV-related hospitalization among people living with HIV

Parameter	Unadjusted		Adjusted^a^	
	HR (95% CI)	p value^d^	aHR (95% CI)	p value^d^
Number of outpatient psychiatric visits^b^				
Reference: 0				
1–2	**1.72 (1.5, 1.97)**	< 0.0001	**1.42 (1.22, 1.65)**	< 0.0001
3–5	**1.88 (1.57, 2.26)**	< 0.0001	**1.58 (1.29, 1.94)**	< 0.0001
6–10	**1.78 (1.44, 2.21)**	< 0.0001	**1.51 (1.18, 1.94)**	0.0010
> 10	**1.71 (1.42, 2.06)**	< 0.0001	**1.76 (1.37, 2.25)**	< 0.0001
			p(trend)^c^ < 0.0001	
Any previous diagnosis^b^ of				
Alcohol-related disorder	**2.16 (1.75, 2.66)**	< 0.0001	**1.40 (1.12, 1.74)**	0.003
Reference: no alcohol-related disorder				
Substance-related disorder	**2.07 (1.82, 2.36)**	< 0.0001	**1.17 (1.00, 1.36)**	0.04
Reference: no substance-related disorder				
Anxiety disorder	**1.21 (1.04, 1.4)**	0.013	1.06 (0.90, 1.26)	0.48
Reference: no anxiety disorder				
Depression disorder	**1.20 (1.05, 1.37)**	0.006	0.93 (0.80, 1.09)	0.36
Reference: no depression disorder				
Assigned female at birth	1.11 (0.99, 1.23)	0.07	**1.23 (1.10, 1.38)**	0.0002
Reference: male				
Race/ethnicity				
Reference: White Non-Hispanic				
Black non-Hispanic	1.19 (0.9, 1.57)	0.22	1.15 (0.87, 1.52)	0.34
Hispanic	1.09 (0.83, 1.44)	0.52	1.01 (0.76, 1.34)	0.95
Other/Unknown	0.9 (0.66, 1.24)	0.53	0.95 (0.69, 1.32)	0.78
Age (in years)	1.02 (1.01, 1.02)	0.16	**1.01 (1.00, 1.01)**	0.007
CD4 T-cell count (cells/uL)				
Reference: ≥500				
< 200	**19.18 (16.08, 22.87)**	< 0.0001	**14.74 (12.21, 17.79)**	< 0.0001
200–349	**4.54 (3.69, 5.59)**	< 0.0001	**3.86 (3.12, 4.78)**	< 0.0001
350–499	**1.56 (1.20, 2.03)**	0.001	**1.46 (1.12, 1.89)**	0.005
Viral load undetectable	**0.25 (0.22, 0.28)**	< 0.0001	**0.57 (0.50, 0.65)**	< 0.0001
Reference: detectable				

Bold indicates statistical significance p ≤ 0.05

*HR* hazard ratio, *aHR* adjusted hazard ratio

^a^Model consisting of all Table 3 predictors (Number of previous outpatient psychiatric visits, types of previous diagnoses, sex assigned at birth, race/ethnicity, age, CD4 T-cell count, viral load)

^b^Time-varying covariate

^c^Test for linear trend across number of previous psychiatric visits

^d^Corresponding to test of nonzero regression coefficient in the stratified Cox model

In adjusted analyses, the hazard of HIV-related hospitalization again was again significantly higher with the any number of previous outpatient psychiatric visits > 0 [1–2 visits, aHR 1.42, 95% CI 1.22–1.65; 3–5 visits, aHR 1.58, 95% CI 1.29–1.94; 6–10 visits, aHR 1.51, 95% CI 1.18–1.94; >10 visits, aHR 1.76, 95% CI 1.37–2.25; p(trend) < 0.0001]. Particular psychiatric diagnoses significantly associated with hazard for HIV-related hospitalizations included alcohol-related disorder (aHR 1.40, 95% CI 1.12–1.74, p = 0.003) and/or substance-related disorder (aHR 1.17, 95% CI 1.00–1.36, p = 0.04). Demographic and clinical factors mirrored those associated with any psychiatric hospitalization. Specifically, female sex (aHR 1.23, 95% CI 1.10–1.38, p = 0.0002) and older age (aHR 1.01 per year, 95% CI 1.00–1.01, p = 0.007) were associated with higher hazard, as were CD4 count < 500 cells/uL (i.e., < 200 cells/uL: aHR 14.74, 95% CI 12.21–17.79, p < 0.0001; 200–349 cells/uL: 3.86, 95% CI 3.12–4.78, p < 0.0001; 350–499 cells/uL: 1.46, 95% CI 1.12–1.89, p = 0.005) and detectable viral load (undetectable viral load aHR 0.57 vs. detectable, 95% CI 0.50–0.65, p < 0.0001).

Again, sensitivity analyses did not meaningfully change results and there was no indication that the observed effects varied across calendar period. Associations of psychiatric, demographic, and HIV-related factors with HIV-related hospitalizations were consistent after imputing missing CD4/viral load data.

## Discussion

Results of the current study show that a considerable proportion of PLWH in our study were hospitalized for non-psychiatric concerns, with a median time to incident hospitalization of 30.8 months. Similarly, a substantial proportion were hospitalized for HIV-related concerns, with a median time to incident HIV-related hospitalization of 54.5 months. Consistent with hypotheses 1–2, results suggest that a history of a higher number of prior outpatient psychiatric visits and a history of depression were associated with increased hazard for any non-psychiatric hospitalization. Consistent with hypothesis 3, demographic characteristics such as being female or older, and HIV-related characteristics such as having a CD4 count < 500 cells/uL were also associated with increased risk for any non-psychiatric hospitalization. Having an undetectable viral load was associated with lower risk.

In terms of HIV-related hospitalization, consistent with hypotheses, results suggest a higher number of previous outpatient psychiatric visits, a past diagnosis of alcohol- or substance-related disorders, and CD4 count < 500 cells/uL or a detectable viral load were associated with increased risk. Female sex and age were associated with increased risk, while race/ethnicity was not. These results suggest that more severe and complex psychiatric disorders, which may require more frequent psychiatric visits, are associated with higher hazard of hospitalization. Patients with increased service utilization may have had greater medical needs due to higher (or more chronic) psychiatric concerns, and/or may have been screened more often and referred to inpatient care.

Patients with depression, substance-related, and alcohol-related disorders seem to be at the most elevated risk. This was particularly evident when comparing unadjusted versus adjusted analyses. In unadjusted analyses, any type of psychiatric diagnostic history (i.e., having a history of an alcohol-related disorder, substance-related disorder, depression, or anxiety) was significantly associated with both outcomes. Depression, the most common psychiatric comorbidity for PLWH with prevalence ranging from 20 to 40% across studies [27], remained associated with any non-psychiatric hospitalization after adjusting for all other factors. Depression has been shown to be associated with myriad adverse health outcomes for PLWH, including lower CD4 count, higher viral load, lower medication adherence, and AIDS-related and all-cause mortality [17, 27, 29]. Behaviorally, depression among PLWH impairs people’s abilities to manage their HIV-related health, in part due to increased avolition and hopelessness [28]. In our study, depression was associated with non-psychiatric outcomes even when CD4 count and viral load are held constant. Depression and alcohol-related disorders may be critical targets for integrated psychiatric and HIV-related intervention.

Results suggest that sex (female) and age (older) are associated with higher risk for hospitalization. Indeed, older women living with HIV are disparately affected by depression, due largely to social stigma and discrimination, inequities in access to healthcare and HIV-related support services, and an unequal burden of physiological effects of aging with the virus (e.g., neuroinflammation, muscle loss, early-onset menopause) [15, 28]. Thus, interventions targeting psychiatric comorbidity may need to address social-cultural factors unique to women living (and aging) with HIV.

In terms of HIV-related hospitalizations, the only types of psychiatric diagnoses that remained significantly associated in adjusted models were alcohol- and substance-related disorders. Similar to depression, alcohol- and substance-related disorders impact medication adherence, accelerate disease progression, and result in spikes in viral load and treatment avoidance/dropout [15]. Indeed, PLWH with comorbid alcohol- and substance-related diagnoses are more likely to utilize emergency services (rather than outpatient preventative medicine) and thus may be more likely to be referred to an inpatient stay [4, 8]. These results suggest a complex interplay between psychiatric comorbidity and medical outcomes for PLWH, whereby particular mechanisms critical to maintaining one’s health while living with HIV are likely profoundly affected by alcohol and substance use. Each of these mechanisms thus likely drive higher need for hospitalization, and thus are critical targets for behavioral and system-level intervention for PLWH who have comorbid depression, alcohol-related, and substance-related disorders. Of note, models were time-stratified, and identified associations were consistent over time, even in recent years. This suggests that high-level changes in HIV-related care (e.g., improvements in medication regimens, healthcare and health insurance policies, and HIV care cascades) did not meaningfully resolve these issues for PLWH.

### Limitations

The study’s findings must be considered in the context of its limitations, particularly the biases inherent in EHR-based secondary data analysis, including that the data were collected retrospectively. Our reliance on billing codes as a proxy for psychiatric and HIV-related health was inherently limited, due to the possibility of provider/human error. Our reliance on hospital billing codes as the sole indicator of patient psychiatric health also threatens internal validity. Billing codes are not always a reliable indicator of psychiatric health or diagnosis, as admitting providers may provide a diagnosis in the absence of a thorough assessment due staffing and time limitations, or to facilitate insurance coverage for patients who do not meet diagnostic criteria. Future retrospective electronic medical record review studies might consider validating diagnoses in a subset using more detailed chart review.

Similarly, the cohort for the current study is limited to patients engaged in care at Montefiore, and thus we cannot report services utilized beyond this healthcare system. It is thus possible that patients in the sample had additional inpatient visits and/or psychiatric diagnoses and treatment not captured within this healthcare system’s EHR. All participants in this study were confirmed to have HIV and thus were receiving care for HIV, biasing the sample to treatment-engaged individuals. Thus, our study’s conclusions may not generalize to a broader population of PLWH who also include those who are not in active care for HIV. Understanding the links between psychiatric diagnosis and risk for hospitalization must therefore be investigated in non-care-seeking PLWH. Our analyses did not control for variations in healthcare system-level differences, as we did not measure visit location, provider, or setting. This may be an important inquiry for future studies to determine which locations, providers, and/or settings patients visit may be associated with increased or decreased likelihood for hospitalization. For a full understanding of service utilization beyond one healthcare system, future studies may integrate EHR data across multiple systems or utilize insurance claims data.

Further, there are inherent limitations of grouping demographic characteristics in EHR data, particularly in categorizing patient sex as binary and categorizing race and ethnicity in fixed, monoracial groups, effectively erasing transgender, nonbinary and multiracial identities. Future studies that integrate patient and provider self-report data (i.e., qualitative interviews, survey data) with routinely collected health record data may tell a more comprehensive story about which intervention targets and opportunities would benefit patients the most.

### Implications for Healthcare Systems

Despite limitations, results echo calls for a comprehensive, integrated response to support mental health and HIV-related health among diverse patient populations. Suggestions for healthcare system interventions include: integrated care, whereby HIV and mental health services are integrated to ensure interdisciplinary providers collaborate to address comorbid symptomatology [12, 29]; and comprehensive case management, wherein healthcare systems may partner with community-based organizations and other local resource groups to provide targeted case management addressing comorbid concerns [19]. Given the study’s findings that more outpatient visits did not prevent hospitalizations, it is critical to go beyond current standards of care and provide integrated, comprehensive supports for diverse groups of PLWH. Healthcare systems may need to increase utilization of adjunctive supportive care, including comprehensive case management services that bridge HIV- and non-HIV-related services to expedite access to life-saving resources for PLWH and psychiatric disorders [19]. Comprehensive case management services can simultaneously address behavioral concerns for PLWH, as well as buffer the impacts of socio-cultural determinants of health by linking patients to housing, food, medication adherence programs, and harm reduction programs for substance and alcohol use [12, 15, 19].

### Behavioral Implications

Results provide further support for studies and interventions that target behavioral and socio-cultural mechanisms accelerating links between mental health and hospitalization. These may include targeting the roles of stigma, sexism, and ageism in the lives of PLWH, especially women and older adults [15, 16]. Further work is needed to identify specific pathways by which behavioral and psychological factors (e.g., treatment adherence, internalized stigma, HIV-related social support) may lead to increased rates of hospitalization in order to bolster material, psychological, and behavioral support for people PLWH.

## Conclusions

These findings provide a fruitful avenue for future clinical inquiry, highlighting questions about how and why different diagnoses confer differential risk for non-psychiatric hospitalizations. Future research is needed to clarify individual-level mechanisms which are driven by social determinants of health. Last, we hope these findings echo the need to develop community-grounded, wraparound interventions to promote positive conditions of living to improve health outcomes for PLWH.

### Supplementary Information

Below is the link to the electronic supplementary material.

Supplementary file1 (DOCX 16 KB)

## Data Availability

Data are held by the data informatics team at the Einstein-Rockefeller-CUNY Center for AIDS Research.
