# Supplementary material for: Longitudinal Associations of Psychiatric Risk Factors with Non-psychiatric Hospitalization in a Large Cohort of People Living with HIV in New York City
Source: AIDS Behav. 2023 Apr 21;27(10):3487–97. doi: 10.1007/s10461-023-04064-6 (PMC10516773; doi:10.1007/s10461-023-04064-6)
Supplement: Supplementary file 1 — Supplementary file1 (DOCX 16 KB) [file 10461_2023_4064_MOESM1_ESM.docx]

| Table S1. ICD-9-CM/ICD-10-CM codes used to identify types of hospitalizations and psychiatric factors | | |
| --- | --- | --- |
| **Description** | **ICD-9-CM code**  **(through 2015)** | **ICD-10-CM code**  **(2015 and later)** |
| HIV-related hospitalization (discharge diagnosis) | 042, 042.0, 042.1, 042.2, 042.9, 043, 043.1, 043.2, 043.3, 043.9, 044, 044.0, 044.9, 079.53, V08 | B20, B97.35, Z21 |
| Alcohol-related disorders | 291.0, 291.1, 291.2, 291.3,  291.4, 291.5, 291.8, 291.81, 291.82, 291.89, 291.9, 303.00, 303.01, 303.02, 303.90, 303.91, 303.92, 305.00, 305.01, 305.02, 357.5, 425.5, 535.30, 535.31, 571.0, 571.1, 571.2, 571.3, 760.71, 980.0, V65.42, V79.1, E860.0 | F10.10, F10.120, F10.121, F10.129, F10.14, F10.150, F10.151, F10.159, F10.180, F10.181, F10.182, F10.188, F10.19, F10.20, F10.220, F10.221, F10.229, F10.230, F10.231, F10.232, F10.239, F10.24, F10.250, F10.251, F10.259, F10.26, F10.27, F10.280, F10.281, F10.282, F10.288, F10.29, F10.920, F10.921, F10.929, F10.94, F10.950, F10.951, F10.959, F10.96, F10.97, F10.980, F10.981, F10.982, F10.988, F10.99, G62.1, I42.6, K29.20, K29.21, K70.0, K70.10, K70.11, K70.2, K70.30, K70.31, K70.40, K70.41, K70.9, P04.3, Q86.0, T51.0X1A, T51.0X2A, T51.0X3A, T51.0X4A, Z13.89, Z71.41, Z71.42, Z71.51, Z71.52, Z71.6 |
| Substance-related disorder | 292.0, 292.11, 292.12, 292.2, 292.81, 292.82, 292.83, 292.84, 292.85, 292.89, 292.9, 304.00, 304.01, 304.02, 304.10, 304.11, 304.12, 304.2, 304.20, 304.21, 304.22, 304.3, 304.30, 304.31,  304.32, 304.4, 304.40, 304.41, 304.42, 304.5, 304.50, 304.51, 304.52, 304.6, 304.60, 304.61, 304.62, 304.7, 304.70, 304.71, 304.72, 304.8, 304.80, 304.81, 304.82, 304.9, 304.90, 304.91,  304.92, 305.2, 305.20, 305.21, 305.22, 305.3, 305.30, 305.31, 305.32, 305.4, 305.40, 305.41, 305.42, 305.5, 305.50, 305.51, 305.52, 305.6, 305.60, 305.61, 305.62, 305.7, 305.70, 305.71, 305.72, 305.8, 305.80, 305.81, 305.82, 305.9, 305.90, 305.91, 305.92, 648.3, 648.30, 648.31, 648.32, 648.33, 648.34, 655.5, 655.50, 655.51, 655.53, 760.72, 760.73, 760.75, 779.5, 965.0, 965.00, 965.01, 965.02, 965.09, V65.42, E850.0, E850.1, E850.2, E854.1, E935.0, E935.1 | F11.10, F11.120, F11.121, F11.122, F11.129, F11.14, F11.150, F11.151, F11.159, F11.181, F11.182, F11.188, F11.19, F11.20, F11.220, F11.221, F11.222, F11.229, F11.23, F11.24, F11.250, F11.251, F11.259, F11.281, F11.282, F11.288, F11.29, F11.90, F11.920, F11.921, F11.922, F11.929, F11.93, F11.94, F11.950, F11.951, F11.959, F11.981, F11.982, F11.988, F11.99, F12.10, F12.120, F12.121, F12.122, F12.129, F12.150, F12.151, F12.159, F12.180, F12.188, F12.19, F12.20, F12.220, F12.221, F12.222, F12.229, F12.250, F12.251, F12.259, F12.280, F12.288, F12.29, F12.90, F12.920, F12.921, F12.922, F12.929, F12.950, F12.951, F12.959, F12.980, F12.988, F12.99, F13.10, F13.120, F13.121, F13.129, F13.14, F13.150, F13.151, F13.159, F13.180, F13.181, F13.182, F13.188, F13.19, F13.20, F13.220, F13.221, F13.229, F13.230, F13.231, F13.232, F13.239, F13.24, F13.250, F13.251, F13.259, F13.26, F13.27, F13.280, F13.281, F13.282, F13.288, F13.29, F13.90, F13.920, F13.921, F13.929, F13.930, F13.931, F13.932, F13.939, F13.94, F13.950, F13.951, F13.959, F13.96, F13.97, F13.980, F13.981, F13.982, F13.988, F13.99, F14.10, F14.120, F14.121, F14.122, F14.129, F14.14, F14.150, F14.151, F14.159, F14.180, F14.181, F14.182, F14.188, F14.19, F14.20, F14.220, F14.221, F14.222, F14.229, F14.23, F14.24, F14.250, F14.251, F14.259, F14.280, F14.281, F14.282, F14.288, F14.29, F14.90, F14.920, F14.921, F14.922, F14.929, F14.94, F14.950, F14.951, F14.959, F14.980, F14.981, F14.982, F14.988, F14.99, F15.10, F15.120, F15.121, F15.122, F15.129, F15.14, F15.150, F15.151, F15.159, F15.180, F15.181, F15.182, F15.188, F15.19, F15.20, F15.220, F15.221, F15.222, F15.229, F15.23, F15.24, F15.250, F15.251, F15.259, F15.280, F15.281, F15.282, F15.288, F15.29, F15.90, F15.920, F15.921, F15.922, F15.929, F15.93, F15.94, F15.950, F15.951, F15.959, F15.980, F15.981, F15.982, F15.988, F15.99, F16.10, F16.120, F16.121, F16.122, F16.129, F16.14, F16.150, F16.151, F16.159, F16.180, F16.183, F16.188, F16.19, F16.20, F16.220, F16.221, F16.229, F16.24, F16.250, F16.251, F16.259, F16.280, F16.283, F16.288, F16.29, F16.90, F16.920, F16.921, F16.929, F16.94, F16.950, F16.951, F16.959, F16.980, F16.983, F16.988, F16.99, F17.203, F17.208, F17.209, F17.213, F17.218, F17.219, F17.223, F17.228, F17.229, F17.293, F17.298, F17.299, F18.10, F18.120, F18.121, F18.129, F18.14, F18.150, F18.151, F18.159, F18.17, F18.180, F18.188, F18.19, F18.20, F18.220, F18.221, F18.229, F18.24, F18.250, F18.251, F18.259, F18.27, F18.280, F18.288, F18.29, F18.90, F18.920, F18.921, F18.929, F18.94, F18.950, F18.951, F18.959, F18.97, F18.980, F18.988, F18.99, F19.10, F19.120, F19.121, F19.122, F19.129, F19.14, F19.150, F19.151, F19.159, F19.16, F19.17, F19.180, F19.181, F19.182, F19.188, F19.19, F19.20, F19.21, F19.220, F19.221, F19.222, F19.229, F19.230, F19.231, F19.232, F19.239, F19.24, F19.250, F19.251, F19.259, F19.26, F19.27, F19.280, F19.281, F19.282, F19.288, F19.29, F19.90, F19.920, F19.921, F19.922, F19.929, F19.930, F19.931, F19.932, F19.939, F19.94, F19.950, F19.951, F19.959, F19.96, F19.97, F19.980, F19.981, F19.982, F19.988, F19.99, F55.0, F55.1, F55.2, F55.3, F55.4, F55.8, O35.5XX0, O35.5XX1, O35.5XX2, O35.5XX3, O35.5XX4, O35.5XX5, O35.5XX9, T40.691A, T40.692A, T40.693A, T40.694A, O99.320, O99.321, O99.322, O99.323, O99.324, O99.325, P04.41, P04.49, P96.1, P96.2, T40.0X1A, T40.0X2A, T40.0X3A, T40.0X4A, T40.0X5A, T40.0X5S, T40.1X1A, T40.1X2A, T40.1X3A, T40.1X4A, T40.2X1A, T40.2X2A, T40.2X3A, T40.2X4A, T40.3X1A, T40.3X2A, T40.3X3A, T40.3X4A, T40.3X5A, T40.3X5S, T40.4X1A, T40.4X2A, T40.4X3A, T40.4X4A, T40.7X1A, T40.8X1A, T40.601A, T40.602A, T40.603A, T40.604A, T40.691A, T40.692A, T40.693A, T40.694A, T40.901A, T40.991A, Z71.41, Z71.42, Z71.51, Z71.52, Z71.6 |
| Anxiety disorder | 293.84, 300.00, 300.01, 300.02, 300.09, 300.10, 300.20, 300.21, 300.22, 300.23, 300.29, 300.3, 300.5, 300.89, 300.9, 308.0, 308.1, 308.2, 308.3, 308.4, 308.9, 309.81, 313.0, 313.1, 313.21, 313.22, 313.3, 313.82, 313.83 | F06.4, F40.00, F40.01, F40.02, F40.10, F40.11, F40.210, F40.218, F40.220, F40.228, F40.230, F40.231, F40.232, F40.233, F40.240, F40.241, F40.242, F40.243, F40.248, F40.290, F40.291, F40.298, F40.8, F40.9, F41.0, F41.1, F41.3, F41.8, F41.9, F42, F43.0, F43.10, F43.11, F43.12, F44.9, F45.8, F48.8, F48.9, F93.8, F99, R45.2, R45.5, R45.6, R45.7 |
| Depression disorder | 296.20, 296.21, 296.22, 296.23, 296.24, 296.25, 296.26, 296.30, 296.31, 296.32, 296.33, 296.34, 296.35, 296.36, 296.51, 296.52, 296.53, 296.54, 296.55, 296.56, 296.60, 296.61, 296.62, 296.63, 296.64, 296.65, 296.66, 296.89, 298.0, 300.4, 309.1, 311 | F31.30, F31.31, F31.32, F31.4, F31.5, F31.60, F31.61, F31.62, F31.63, F31.64, F31.75, F31.76, F31.77, F31.78, F31.81, F32.0, F32.1, F32.2, F32.3, F32.4, F32.5, F32.9, F33.0, F33.1, F33.2, F33.3, F33.40, F33.41, F33.42, F33.9, F34.1, F43.21 |
